# MDI versus CSII in Chinese adults with type 1 diabetes in a real-world situation: based on propensity score matching method

**DOI:** 10.1186/s12955-024-02263-w

**Published:** 2024-06-13

**Authors:** Jian Yu, Hong Wang, Min Zhu, Jingjing Xu

**Affiliations:** 1 Department of Endocrinology, the First Affiliated Hospital with Nanjing Medical University(Jiangsu Province Hospital), 300 Guangzhou Road, Nanjing, LA 210029 China; 2 Department of Nursing, the First Affiliated Hospital with Nanjing Medical University(Jiangsu Province Hospital), Nanjing, China

**Keywords:** Adults, Fear of hypoglycemia, Glycated hemoglobin, Insulin injection, Quality of life, Type 1 diabetes

## Abstract

**Background:**

Compared with multiple daily insulin injections (MDI), continuous subcutaneous insulin infusion (CSII) is significantly more expensive and has not been widely used in Chinese type 1 diabetes mellitus (T1DM) patients. So there are still significant knowledge gaps regarding clinical and patient-reported outcomes in China.

**Aims:**

This study aims to compare the glycated hemoglobin (HbA_1C_), insulin therapy related quality of life (ITR-QOL), fear of hypoglycemia (FOH) of adult T1DM patients treated with MDI and CSII based on propensity score matching in real-world conditions in China.

**Methods:**

Four hundred twenty adult T1DM patients who were treated with MDI or CSII continuously for more than 12 months in a national metabolic center from June 2021 to June 2023 were selected as the study subjects. Their QOL and FOH were evaluated with Insulin Therapy Related Quality of Life Measure Questionnaire-Chinese version (ITR-QOL-CV) and the Chinese Version Hypoglycemia Fear Survey-Worry Scale (CHFSII-WS), and their HbA_1C_ were collected at the same time. Potential confounding variables between the two groups were matched using propensity score matching.

**Results:**

Of the 420 patients included in the study, 315 were in MDI group and 105 were in CSII group. 102 pairs were successfully matched. After matching, the total score of ITR-QOL-CV scale in CSII group was significantly higher than that in MDI group (87.08 ± 13.53 vs. 80.66 ± 19.25, *P* = 0.006). Among them, the dimensions of daily life, social life, and psychological state were all statistically different (*P* < 0.05). The scores of CHFSII-WS (8.33 ± 3.49 vs. 11.77 ± 5.27, *P* = 0.003) and HbA_1C_ (7.19 ± 1.33% vs. 7.71 ± 1.93%, *P* = 0.045) in CSII group were lower than those in MDI group.

**Conclusions:**

25.0% of T1DM adults are treated with CSII. Compared with adult T1DM patients treated with MDI, those treated with CSII have higher ITR-QOL, less FoH, and better control of HbA_1C_ in real-world conditions in China. Therefore, regardless of economic factors, CSII is recommended for adult T1DM patients to optimize the therapeutic effect and outcomes.

## Background

Type 1 diabetes mellitus (T1DM) is a chronic disease mediated by autoimmune impaired islet β cells, leading to severe endogenous insulin deficiency [1]. Despite the younger peak age of the onset of T1DM, new-onset T1DM occurs in all age-groups and people with T1DM live for many decades after the onset of the disease, such that the overall prevalence of T1DM is higher in adults than in children, justifying our focus on T1DM in adults [2, 3]. Due to the absolute lack of self-insulin secretion, T1DM patients require exogenous insulin replacement to control blood glucose. Currently, multiple daily insulin injections (MDI) or continuous subcutaneous insulin infusion (CSII) are the most important treatment regimen for patients with T1DM worldwide [4].

Both MDI and CSII can optimize the glycaemic control to a near normal level of T1DM patients [1]. Glycated hemoglobin (HbA_1C_) has become the standard biomarker of assessing long-term glycaemic control in patients with diabetes and correlates with the development of complications [5, 6]. A randomized controlled trial in the UK found that during the first year following T1DM diagnosis, no HbA_1C_ benefit of CSII over MDI was identified in children and young people [7]. While a meta-analysis showed that effect of CSII over MDI on HbA_1C_ was − 0.42[− 0.66; −0.18]% in those enrolling only adult T1DM patients [8]. Different study designs and settings may account for this discrepancy.

Considering the enormous daily management burden that T1DM places on patients, benefits for quality of life (QOL) were afforded equal priority to improvements in HbA_1C_ in the past decades [9]. Adult T1DM patients face the pressure of work, social and family, no matter which injection regimen, daily insulin injection and poor blood glucose control will bring physical and psychological burden to patients, which will greatly affect their QOL. A previous review study has shown that CSII users have a lower QOL because of disease exposure, the potential dysfunction of insulin pumps, and the difficulties that CSII users encounter during sexual activity [10]. While another cross-sectional study showed that CSII users scored statistically, significantly better on the satisfaction treatment subscale of the Diabetes Quality of life Brief Clinical Inventory [11]. It is likely that differences in results are due to heterogeneity in study design, sample size, and selection, as well as variation in questionnaires used to assess QOL.

Patients receiving intensive insulin therapy have a significantly higher risk of developing hypoglycemia than those receiving other types of treatment [3, 12]. The physical discomfort experience (dizziness, palpitation, etc.) and the potential threat to life (loss of consciousness, convulsions, etc.) can lead to the fear of hypoglycemia (FOH). FOH has been reported to occur in as many as 44–77% of persons with T1DM [13, 14]. Fear of hypoglycemia often leads to excessive avoidance behaviors such as excessive food intake and self-reduction of insulin dose, which worsen glycemic control, thus leading to complications or aggravating their development [15]. In addition, for adults with T1DM, FOH may also threaten their ability to work and drive. To date, few studies have compared differences in FOH among adults with T1DM using different regimens.

Compared with MDI, CSII is significantly more expensive and has not been widely used in China [16]. Hence, there are still significant knowledge gaps regarding clinical outcomes and patient-reported outcomes in China, particularly for adult T1DM. Existing studies have predominantly focused on children and adolescents with T1DM, leaving a dearth of research on the adult population [7, 17, 18]. In addition, a systematic review has reported that existing literature on QOL benefits associated with CSII use is limited, with conflicting, often ambiguous results and many design/methodological flaws [19].

The imbalance of potential confounding variables between MDI and CSII groups can distort the relationship between treatment and outcomes, which may lead to certain biases in the study results. For example, Al Shaikh A et al. encouraged more equal gender distribution in future studies for more comprehensive findings while assess the QOL of children with diabetes who use CSII and MDI treatment [17]. The imbalance of potential confounding variables between the treatment groups can distort the relationship between treatment and outcome. Propensity score matching is one, increasingly utilized, method to help account for such imbalances, allowing for a more accurate estimation of the influence of treatment on outcomes in real-world conditions [20]. This method can balance observed covariates between two groups in nonrandomized studies so that the groups are comparable in the sense that their baseline covariates have similar distribution [21]. Therefore, the aim of this study is to compare the differences in HbA_1C_, insulin therapy related quality of life (ITR-QOL), and FOH between MDI and CSII groups effectively by controlling for selection bias through propensity score matching, so as to provide a basis for guiding adult T1DM patients to choose the appropriate insulin treatment in China.

## Methods

### Patients and study design

Four hundred twenty adult T1DM patients meeting the inclusion criteria were admitted to the endocrinology department of a national metabolic center from June 2021 to June 2023 were included in this study. Inclusion criteria: Patients diagnosed with T1DM and aged over 18 years were eligible to participate; receive MDI of subcutaneous basal insulin analogs and mealtime rapid-acting insulin analogs via insulin pen, or CSII of a rapid-acting insulin analog via a pump, delivered as continuous basal insulin combined with manual mealtime boluses to control their blood glucose for more than 12 months. Exclusion criteria: (1) Patients who have changed their insulin injection regimen in the past 12 months or who were also prescribed with non-insulin blood sugar control drugs (a glucagon-like peptide-1 agonist or any other oral medication) at the same time; (2) Patients with severe acute complications, such as acute infection and diabetic ketoacidosis; (3)Patients with anemia or other factors that may affect HbA_1C_ results; (4) Patients with other serious chronic diseases (such as tumors) that may affect their QOL. This study was approved by the Ethics Committee of the First Affiliated Hospital of Nanjing Medical University (2019-SR-268) and conducted in accordance with the Declaration of Helsinki. All the patients included in this study signed the informed consent form.

### Data collection

1. Sociodemographic and Clinical Variables: Two fixed diabetes education nurses with professional training consulted the inpatient medical records of all patients and extracted their demographic and sociological data, including age, gender, body mass index (BMI), education level, employed or not, etc. Disease-related data, including duration of disease, insulin injection regimen, and with diabetic chronic complications or not. After collection, the relevant data is verified again with the patient to ensure that all data is correct.
2. HbA_1C_: All HbA_1C_ results were obtained from the medical record at the same time, and the cut-off for optimal glycaemic control was set at ≤ 7.0% [2].
3. Insulin Therapy Related Quality of Life Measure Questionnaire -Chinese version (ITR-QOL-CV) : This study adopted the ITR-QOL-CV developed by Ishii et al., [22] which was translated by Chinese scholar Liu Weiwei et al. with Cronbach’s α coefficient of 0.89 [23]. ITR-QOL-CV is a reliable tool for medical staff to evaluate QOL of patients receiving insulin therapy. The 23 main items of the scale include 4 dimensions: daily life (6 items), social activities (6 items), psychological state (9 items), and adverse insulin reactions (2 items). The Likert 5-level scoring method was adopted for the scale, with a total score of 23–115 points. The higher the score, the higher the patient’s insulin therapy related QOL. In this study, Cronbach’α coefficient of ITR-QOL-CV was 0.857.
4. The Chinese Version Hypoglycemia Fear Survey-Worry Scale (CHFSII-WS): This study adopted the CHFSII-WS developed by Professor Cox DJ et, al. from the University of California Health Science Center [24], which was translated into Chinese by scholars such as Mu Chun et al. with a Cronbach’s α of 0.904 [25]. It is a specific tool for medical staff to evaluate patients’ FOH. The 13 items of the scale can be divided into two dimensions: worry and fear (10 items) and awkward emotions (3 items). Each item is scored on a 0–4 scale based on the patient’s feelings in the past 6 months, with a score ranging from 0 to 52. The higher the total score, the higher the level of fear of hypoglycemia. In this study, Cronbach’α coefficient of CHFSII-WS was 0.844.

### Sample size

The overall sample size calculation was conducted using PASS 2021 software (UT, U.S.A) using a two correlated proportions in a matched case-control design. With α = 0.05, a power of 0.90, and an odds ratio = 3.0. We then calculated that at least 76 patients should be enrolled in each group (MDI and CSII group). To ensure an adequate sample size after matching, we plan to include at least 100 patients with CSII.

### Statistical analysis

Continuous data with normal distribution were represented by mean (SD), and the comparison between groups was performed by independent sample *t* test. Continuous data with non-normal distribution were expressed as *M* (Q1, Q3), and the comparison between groups was performed using Mann-Whitney U test. Categorical data were represented by the number of cases or rates, and the comparison between groups was performed by Chi-square test.

The extended program for propensity score matching achieves propensity score matching between MDI and CSII groups, using a 1:1 proximity matching method. The matching procedure was performed using the nearest neighbor method without replacement and with a caliper width of 0.2 of the pooled standard deviation of the logit of the propensity score.

All statistical analyses were performed with SPSS version 26.0 (IBM Corp., Armonk, NY, USA). *P* values ≤ 0.05 were considered statistically significant.

## Results

### Characteristics of the patients before and after matching

**Fig. 1:**
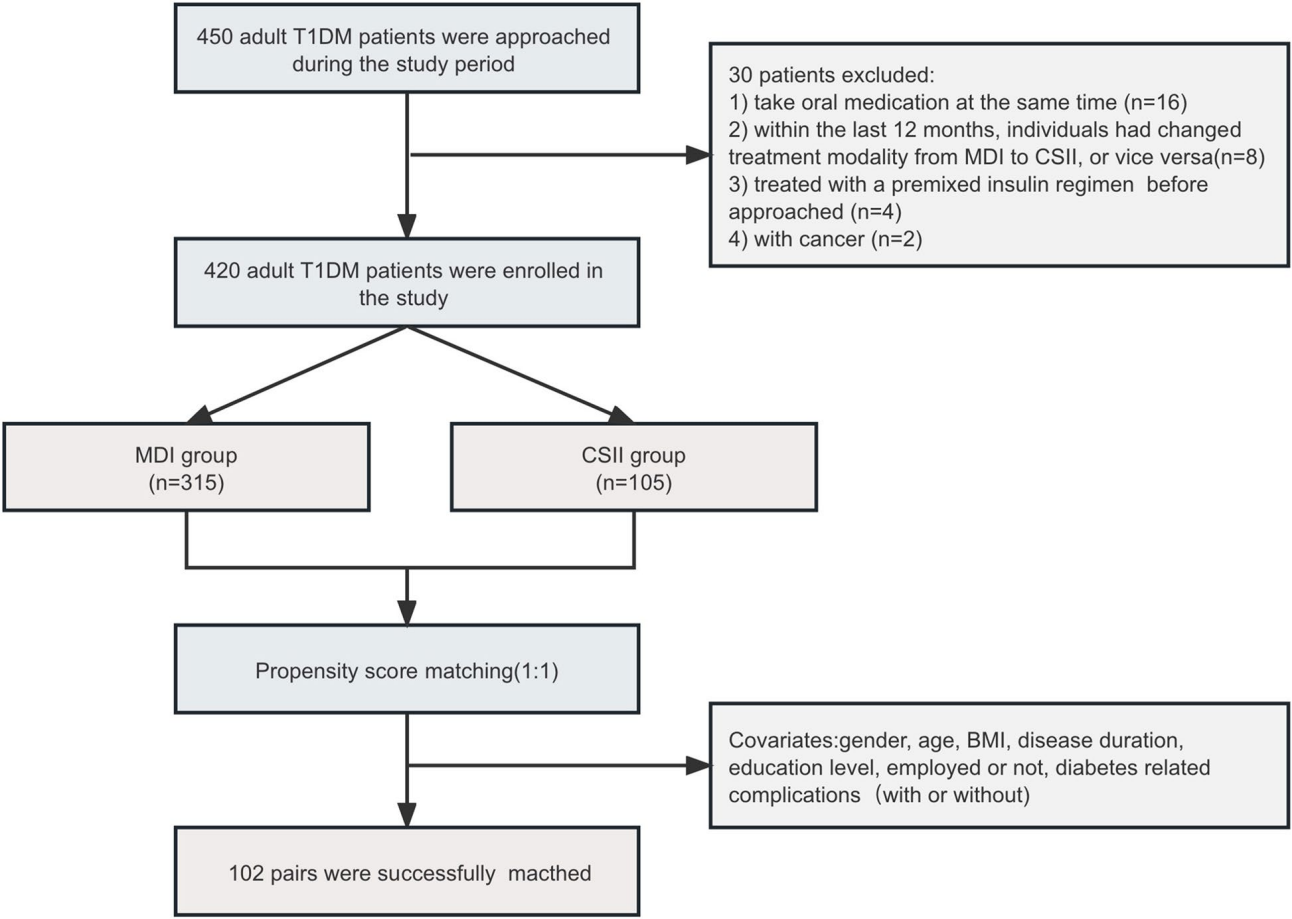
Flowchart of patient recruitment

Four hundred twenty adult T1DM patients were included in this study, including 315 patients treated with MDI and 105 patients treated with CSII. By insulin injection (MDI vs. CSII) is the grouping variable, using the general conditions (including gender, age, BMI, disease duration, education level, employed or not, with diabetes related complications or not) as the Logistic regression analysis. 102 pairs of patients were matched by the nearest neighbor distance matching of the propensity score (Fig. 1.). The characteristics of the two groups of patients before and after matching are shown in Table 1.

**Table 1:** Characteristics of patients before and after the propensity score matching analysis

Parameter	Before matching				After matching			
	MDI (*n* = 315)	CSII (*n* = 105)	t /χ²/Z	*P* value	MDI (*n* = 102)	CSII (*n* = 102)	t /χ²/Z	*P* value
**Age, mean (SD), years**	40.9 (13.3)	38.4 (12.5)	-1.725	**0.085**	37.4 (12.4)	38.3 (12.6)	0.656	0.513
**Gender, n(%)**								
Male	148 (46.98)	36 (34.29)	5.158	**0.023**	38 (37.25)	38 (37.25)	-	-
Female	167 (53.02)	69 (65.71)			64 (62.75)	64 (62.75		
**BMI, mean (SD), kg/m** ^**2**^	21.34 (2.59)	21.00 (2.78)	-1.125	0.261	21.47 (2.90)	20.93 (2.80)	-1.305	0.193
**Disease Duration, M (P25, P75), years**	4.5 (1.6,10.4)	8.4 (2.9,17.4)	-3.813	**< 0.001**	7.1 (3.0,14.0)	8 (2.8,16.1)	-0.843	0.399
**Education level, n (%)**								
Primary school	10 (3.18)	7 (6.67)	8.612	**0.013**	4 (3.92)	7 (6.86)	1.677	0.432
Middle school	137 (43.49)	30 (28.57)			37 (36.28)	30 (29.41)		
College or above	168 (53.33)	68 (64.76)			61 (59.80)	65 (63.73)		
**Employed, n(%)**								
Yes	231 (73.33)	78 (74.29)	0.037	0.848	77 (75.49)	75 (73.53)	0.103	0.748
No	84 (26.67)	27 (25.71)			25 (24.51)	27 (26.47)		
**Diabetic complications, n (%)**								
With	123 (39.05)	54 (51.43)	4.951	**0.026**	43 (42.16)	52 (50.98)	1.596	0.207
Without	192 (60.95)	51 (48.57)			59 (57.84)	50 (49.01)		

*Abbreviations*: *MDI M*ultiple daily insulin injection, *CSII C*ontinuous subcutaneous insulin infusion, *BMI *Body mass index

### Comparison of HbA_1C_ between the matched groups

**Fig. 2:**
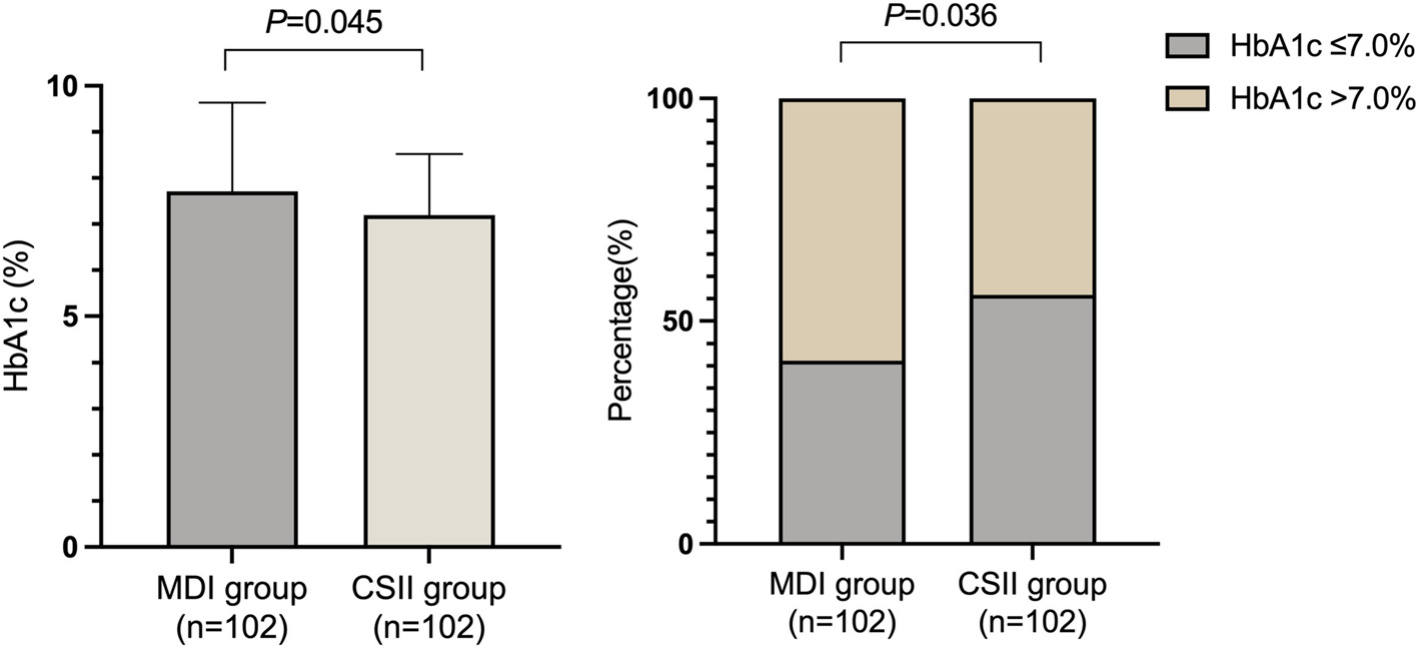
Comparison of HbA1c between the matched groups

The mean HbA_1C_ in the CSII group was 7.19 ± 1.33%, and the mean HbA_1C_ in the MDI group was 7.71 ± 1.93%, with statistical significance (*P* = 0.045). 42 (41.18%) patients in MDI group had their HbA_1C_ less than 7.0% while 57 (55.88%) patients in CSII group had optimal glycaemic control, with statistical significance (*P* = 0.036) (Fig. 2.).

### Comparison of ITR-QOL, FoH between the matched groups

The total score of ITR-QOL-CV in CSII group was significantly higher than that in MDI group (87.08 ± 13.53 vs. 80.66 ± 19.25, *P* = 0.006), among which, the scores of daily life dimension, social activities dimension, and psychological state dimension had statistical differences (*P* < 0.05). The score of CHFSII-WS in CSII group was significantly lower than that in MDI group (8.33 ± 3.49 vs.11.77 ± 5.27, *P* < 0.05). (See Table 2 for details).

**Table 2:** Comparison of ITR-QOL-CV and CHFSII-WS scores between the matched groups

Parameters	MDI (*n* = 102)	CSII (*n* = 102)	t	*P* value
**ITR-QOL-CV scores, mean (SD)**	80.66 (19.25)	87.08 (13.53)	2.76	**0.006**
Daily life (Dimension 1)	19.15 (5.22)	21.64 (4.32)	3.71	**< 0.001**
Social activities (Dimension 2)	22.24 (6.26)	23.78 (4.51)	2.03	**0.04**
Psychological state (Dimension 3)	31.85 (7.60)	34.25 (5.83)	2.52	**0.01**
Adverse insulin reactions (Dimension 4)	7.42 (2.27)	7.41 (1.75)	-0.04	0.97
**CHFSII-WS scores, mean (SD)**	11.77 (5.27)	8.33 (3.49)	-2.98	**0.003**

*Abbreviations*: *MDI *Multiple daily insulin injection, *CSII *Continuous subcutaneous insulin infusion, *ITR-QOL-CV *Insulin Therapy Related Quality of Life Measure Questionnaire-Chinese version, *CHFSII-WS *The Chinese Version Hypoglycemia Fear Survey-Worry Scale

## Discussion

At present, MDI and CSII are the first choice for intensive insulin injection therapy for T1DM patients. Our study found that 25.0% (105/420) of adults with T1DM are treated with CSII and those treated with CSII have better control of HbA_1C,_ higher ITR-QOL, and less FoH in real-world conditions in China.

HbA_1C_ is a classic indicator of the glycemic control of diabetic patients, which can effectively predict the long-term prognosis of patients [26]. The results of this study showed that the blood glucose control of T1DM adults was still far from satisfactory and the HbA_1C_ was better in CSII group than in MDI group in a real-world situation in China. Previous research suggested that in patients with a higher HbA1c levels, a greater reduction in HbA1c levels after CSII occurs [27]. According to the meta-analysis of the three studies included by William et al. [28]., there was no significant difference in the control of HbA_1C_ and time in range in T1DM patients using MDI (72 patients) and CSII (78 patients), but the sample size of each of the above studies was small. In addition, all patients in the study were combined with real-time continuous glucose monitoring. Considering that continuous glucose monitoring can provide patients with more accurate, real-time and intuitive blood glucose information, and patients can adjust diet or insulin dosage in time to correct abnormal blood glucose, the difference in blood glucose control between the two groups may be narrowed. At present, there are few patients applying continuous glucose monitoring in China. In the future, the sample size can be further accumulated to clarify the differences between the two groups of patients in time in range and other blood glucose control indicators.

This study found that compared with MDI group, patients in the CSII group had a higher ITR-QOL, among which the scores of the dimensions of daily life, social activities and psychological state had statistical differences. Al Shaikh A et al. also found children treated with CSII had statistically significant better symptom control, less treatment difficulties, and a higher QOL [17]. CSII allows the administration of additional boluses if needed, with minimal patient discomfort [29]. Patients using a pump have more flexible possibilities regarding meals, diet, everyday activities, and socialization [30]. In addition, CSII can also reduce the pain and inconvenience caused by multiple subcutaneous injections to patients, and it is more convenient for the correction of high and low blood sugar [31]. Therefore, it can effectively reduce the impact on their QOL, which is similar to the findings of Thabit et al. [32] However, another study found that due to the high economic cost of patients in the CSII group, the QOL of patients would decline [33]. One possible reason could be that the EuroQol 5-level 5-dimension questionnaire used in the study is a universal Quality of Life scale that may not be targeted for measuring the changes in QOL in diabetic patients due to insulin injections. In this study, there was no obvious difference in the adverse reaction dimension in the ITR-QOL-CV scale between the two groups. It may be that the adverse reactions of insulin injection are more related to the drug, whether the patient has an allergic constitution, or whether the insulin injection process is standardized.

Intensive insulin therapy increases the risk of hypoglycemia while maintaining normal blood glucose in T1DM patients [34]. Previous studies have shown that FOH is related to the frequency of hypoglycemia, especially severe hypoglycemia [35, 36]. In our study, only 19 of 420 patients (4.52%) wore continuous glucose monitoring on a daily basis. Considering that there are few adult T1DM patients routinely using continuous glucose monitoring in China, it is difficult to effectively capture the true incidence of hypoglycemic events in this population. Therefore, this study used patients’ self-reported FOH scale for relevant evaluation. Studies have demonstrated that FOH may lead to perceived concerns of a mismatch between food intake, insulin dose, or physical activity, resulting in over or under-compensatory behaviors, and can place great mental burden on patients with T1DM [14, 37, 38]. In our study, the FOH of patients in CSII group was lower than that in MDI group. Gomez-Peralta et al. [26] found that patients using CSII have a lower frequency of hypoglycemia than MDI, more hypoglycemia experience may be one of the reasons for the higher FoH in MDI patients. Therefore, more attention should be paid to the evaluation of FOH in T1DM patients with MDI. In practice, newer technologies and individualized strategies to reduce FOH while maintaining optimal glucose control are needed [39]. Besides, Rossi et al. found that hypoglycemia may negatively affect patient QOL [40], further research is needed to explore this relationship in Chinese population.

A strength of our study is the use of to match the confounders of patients between groups, so as to avoid bias in this study. One of the limitations of this study is that only patients with traditional insulin pen and tubular insulin pump were included in this study. With the promotion of needle-free syringe and closed-loop insulin pump system, more patients with different insulin injection methods can be included in the future, so as to enrich relevant research results. In addition, although the evidence of HbA_1C_ reduction remains the most robust measure associated with chronic diabetes complications, more recent studies have begun to examine the relationship between TIR and long-term complications and have provided the basis for glycemic targets with newer glucose monitoring technologies [41].

## Conclusions

This study balanced the confounding factors between the two groups by means of propensity score matching, and scientifically and reliably compared the HbA_1C_, ITR-QOL, and FOH of adult T1DM patients treated with CSII and MDI. Our study found that compared with adult T1DM patients treated with MDI, those treated with CSII have higher ITR-QOL, less FoH, and better control of HbA_1C_ in real-world conditions in China. Therefore, regardless of economic factors, CSII is recommended for adult T1DM patients to optimize the therapeutic effect and outcomes.

## Data Availability

No datasets were generated or analysed during the current study.

## Abbreviations

MDI: Multiple daily insulin injection
CSII: Continuous subcutaneous insulin infusion
T1DM: Type 1 diabetes mellitus
QOL: Quality of life
FOH: Fear of hypoglycemia
HbA_1C_: Glycated hemoglobin
BMI: Body mass index
ITR-QOL-CV: Insulin Therapy Related Quality of Life Measure Questionnaire-Chinese version
CHFSII-WS: The Chinese Version Hypoglycemia Fear Survey-Worry Scale
